# Human Pavlovian fear conditioning conforms to probabilistic learning

**DOI:** 10.1371/journal.pcbi.1006243

**Published:** 2018-08-31

**Authors:** Athina Tzovara, Christoph W. Korn, Dominik R. Bach

**Affiliations:** 1 Clinical Psychiatry Research, Department of Psychiatry, Psychotherapy, and Psychosomatics, University of Zurich, Zurich, Switzerland; 2 Neuroscience Centre Zurich, University of Zurich, Zurich, Switzerland; 3 Wellcome Centre for Human Neuroimaging and Max Planck UCL Centre for Computational Psychiatry and Ageing, University College London, London, United Kingdom; 4 Helen Wills Neuroscience Institute, UC Berkeley, Berkeley, California, United States of America; 5 Institute for Systems Neuroscience, University Medical Center Hamburg-Eppendorf, Hamburg, Germany; Harvard University, UNITED STATES

## Abstract

Learning to predict threat from environmental cues is a fundamental skill in changing environments. This aversive learning process is exemplified by Pavlovian threat conditioning. Despite a plethora of studies on the neural mechanisms supporting the formation of associations between neutral and aversive events, our computational understanding of this process is fragmented. Importantly, different computational models give rise to different and partly opposing predictions for the trial-by-trial dynamics of learning, for example expressed in the activity of the autonomic nervous system (ANS). Here, we investigate human ANS responses to conditioned stimuli during Pavlovian fear conditioning. To obtain precise, trial-by-trial, single-subject estimates of ANS responses, we build on a statistical framework for psychophysiological modelling. We then consider previously proposed non-probabilistic models, a simple probabilistic model, and non-learning models, as well as different observation functions to link learning models with ANS activity. Across three experiments, and both for skin conductance (SCR) and pupil size responses (PSR), a probabilistic learning model best explains ANS responses. Notably, SCR and PSR reflect different quantities of the same model: SCR track a mixture of expected outcome and uncertainty, while PSR track expected outcome alone. In summary, by combining psychophysiological modelling with computational learning theory, we provide systematic evidence that the formation and maintenance of Pavlovian threat predictions in humans may rely on probabilistic inference and includes estimation of uncertainty. This could inform theories of neural implementation of aversive learning.

## Author summary

Using environmental cues to predict threat is a crucial skill, encountered in many species. A laboratory model to study associating predictive cues with aversive events is Pavlovian fear conditioning. A computational understanding of this process at a systems-level is lacking. Here, we investigate which computational learning model best predicts activity of the human autonomic nervous system during fear conditioning. We show, across three data sets and two autonomic readouts, that a probabilistic learning model explains the data decisively better than previously proposed models. We suggest that humans learn to predict threat by maintaining and constantly updating a probabilistic model of the environment through Bayes’ rule.

## Introduction

Learning to predict threat from environmental cues is a skill found in many species across the animal kingdom. A laboratory example is Pavlovian threat conditioning (also termed fear conditioning [1]) in which the contingent presentation of predictive cues (conditioned stimuli, CS) and aversive events (unconditioned stimuli, US) engages a process of associative learning [2]. In mammals, including humans, establishing CS/US associations requires synaptic plasticity in basolateral and central amygdala [2–5] and thus relies on a neural circuit distinct from that involved in learning reward associations [6]. Despite progress in elucidating systems-level mechanisms that pre-process and relay CS and US information to the amygdala [7,8], a computational understanding of aversive learning remains incomplete, and available neural data do not fully fit standard learning models [4,9,10]. Associative learning theory offers a range of computational models, which make specific behavioural predictions. However, many of these models have historically been developed to capture behavioural and neural phenomena in reward learning [11], and it is not known to what extent threat learning follows the same algorithms. Here, we sought to arbitrate between different associative learning models in humans by comparing their trial-by-trial predictions to the measured trajectory of ANS responses, and examine which learning quantities are reflected on different ANS output. We considered models that learn transition probabilities (i.e. parameters) in a known environmental structure, rather than learning the structure itself [10] or its underlying latent causes [12].

Previous models proposed to explain Pavlovian threat learning are derived from classical reinforcement learning (RL) theory and build on Rescorla-Wagner (RW) [13] and Pearce-Hall (PH) [14] rules. Other studies suggested that a combination of these models best captures aversive learning [15,16] (hybrid model, HM). All of these models provide point estimates of future outcomes, by modifying current predictions with a prediction error, weighted by a learning rate that is either fixed (RW) or determined by a fixed proportion of previous prediction errors (PH and HM). While such models can, in theory, be implemented in simple neural architectures [17], at least in the reward domain they have been challenged because they cannot explain some experimental observations such as latent inhibition [18]. However, latent inhibition is easily accommodated by models in which the learning rate is variable and determined by an estimate of the prediction uncertainty [18]. This uncertainty can be implemented in explicit probabilistic computations, for example in hierarchical models [19], or by Kalman filters in the RL framework, which could be classified as implicitly probabilistic and where uncertainty is encoded in a summary statistic, the Kalman gain [18]. Here, we sought to compare previously proposed algorithms of threat learning with a probabilistic account.

Notably, probabilistic RL algorithms [18] and general-purpose, hierarchical probabilistic models [19] contain parameters that are difficult to constrain from biological or psychological principles and thus have to be estimated from measured data. We reasoned that this may be difficult given the relatively low signal-to-noise ratio in ANS estimates. However, if threat learning is adaptive and approximates statistical optimality [20], these parameters could be usefully constrained. This is why we relied on a parameter-free probabilistic model constructed from statistical principles, rather than from neural or cognitive considerations. Specifically, from the perspective of an agent that is fully informed about the task structure, and assumes stationary transition probabilities and trial independence, US occurrence follows a Bernoulli process with a US probability parameter. The model then computes the likelihood over this parameter based on the evidence that has been observed so far. The binomial likelihood takes the functional form of a beta distribution. Hence, we implemented a sequentially updated beta-binomial model [21].

The most common way to behaviourally assess human threat learning is by measuring ANS responses, such as skin conductance responses (SCR, mostly sympathetic) [22] or pupil size responses (PSR, sympathetic and parasympathetic) [23]. These are mediated by neural circuits including the central amygdala and thus directly linked to circuits involved in formation of CS/US associations [24,25]. Here, we optimized signal-to-noise ratio with a statistical framework for psychophysiological modelling (PsPM) [26] that exploits the entire time-series of autonomic measurements rather than a low number of arbitrarily selected data features (e.g. peak-to-trough measurements). We have previously shown that this method allows estimating the ANS input causing SCR [27] and PSR [28] with higher precision than standard approaches.

Crucially, the observation function that maps an associative learning model onto measured behaviour is not known. Unlike choice data, it has been suggested that ANS outputs may not reflect outcome expectations [15,16]. Furthermore, given their distinct innervation it is possible that different ANS outputs may reflect different aspects of the same learning mechanism. Hence, we additionally sought to clarify this observation function for both measures. We hypothesised that PSR and SCR follow the same underlying learning algorithm but might link to this algorithm by different observation functions. Specifically, SCR are known to habituate during learning, and intraneural stimulations suggested that this habituation does not occur in the effector organ [29], such that either the learning model or the observation function needs to account for this phenomenon.

## Results

Independent samples of participants completed three discriminant threat learning experiments in which a CS+ was reinforced in 50% of trials with an electric shock as US, while a CS- was never reinforced. Participants were not instructed about the contingencies, or their stationarity. Two experiments implemented delay conditioning (i.e. CS and US co-terminate) with visual (experiment 1) and auditory (experiment 3) CS, and one was a trace conditioning paradigm (i.e. CS and US do not co-occur) with visual CS (experiment 2) (Table 1). We analysed data from non-reinforced trials (80 CS-, 40 CS+) and estimated the amplitudes of CS-evoked sudomotor input causing SCR (experiment 1–3) and pupillomotor input causing PSR (experiment 3) with established psychophysiological modelling methods [30,31]. Data from an independent experiment were used to optimise SCR analysis (control experiment).

**Table 1:** Description of participants taking part in the four experiments.

Experiment (Dataset code)	Number of all participants	Number of female participants	Age (mean ± s.e.m.)	Modality	Number of excluded participants
**1 (FR)**	34	23	23.3 ± 0.6	SCR	11 (electrode detachment / malfunctioning of the recording system / absence of US)
**2 (TC)**	23	10	23.8±3.0	SCR	5 (electrode detachment / absence of US)
**3 (PubFe)**	22	15	26.4 ± 5.2	SCR	3 (electrode detachment / malfunctioning of recording system)
				PSR	4 (electrode detachment / malfunctioning of recording system)
**Control (DoxMemP)**	23	19	25.6 ± 0.9	SCR	4 (electrode detachment / malfunctioning of the recording system)

We first confirmed that participants learned the CS-US associations. We considered single trial SCR or PSR, and computed linear mixed effects models, with fixed factors CS (CS+/-) and trial, and participant as a random factor. We observed a main effect of CS, i.e. higher anticipatory SCR and PSR amplitude in response to CS+ compared to CS- for each of the three experiments. Additionally, we observed a main effect of trial for all SCR experiments, but not for PSR. None of our datasets showed a CS x Trial interaction after correcting for multiple comparisons (Table 2, S2 Fig). Without correction we observed an interaction for two out of three SCR datasets (F(1, 2139) = 5.09, p = 0.024 and F(1, 2258) = 5.73, p = 0.017 for experiments 2 and 3 respectively), suggesting weak evidence for a decay in CS difference over trials.

**Table 2:** Results of linear mixed effects models, for each of the 3 experiments. Significant effects after correction for multiple comparisons are shown in bold font.

	Df	CS		Trial		CS x Trial	
		F	p	F	p	F	p
**Exp 1: SCR**	1, 2734	**71.17**	**<0.0001**	**471.03**	**<0.0001**	2.59	0.11
**Exp 2: SCR**	1, 2139	**89.29**	**<0.0001**	**151.75**	**<0.0001**	5.09	0.024
**Exp 3: SCR**	1, 2258	**51.98**	**<0.0001**	**237.40**	**<0.0001**	5.73	0.017
**Exp 3: PSR**	1, 2139	**261.36**	**<0.0001**	2.53	0.112	0.30	0.582

**Fig 1:**
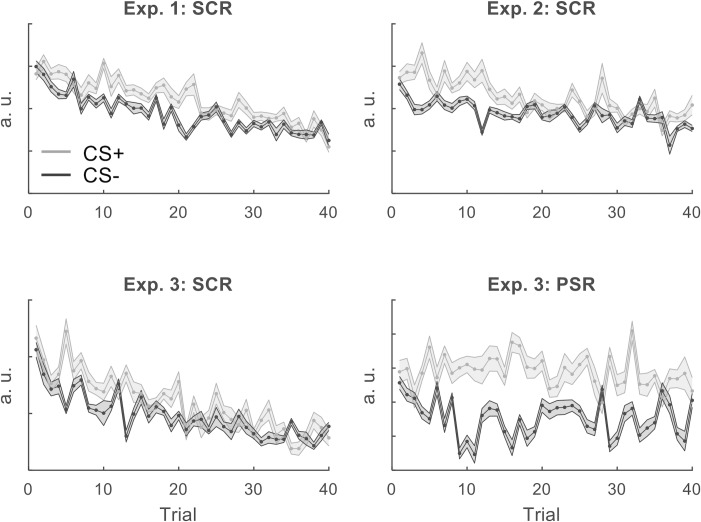
Single trial estimates for SCR and PSR for three experiments, for CS+ / CS- trials. The estimates are derived from psychophysiological models (PsPMs) for anticipatory SCR and PSR. As in our analyses, only US- trials are shown. Because there are twice as many CS- (dark gray) than CS+US- (light gray) trials, we only display CS- responses for every second CS- trial to simplify presentation, but include all CS- responses in our analyses. Shaded bars reflect the standard error across participants.

These findings confirmed our qualitative inspection of the trial-by-trial SCR estimates (Fig 1) and were consistent with previous reports showing a gradual decrease of SCR amplitude over the time-course of the experiment [15,16], while PSR amplitude was sustained (Fig 1). This already suggests that the SCR and PSR may be different.

### Model space

**Fig 2:**
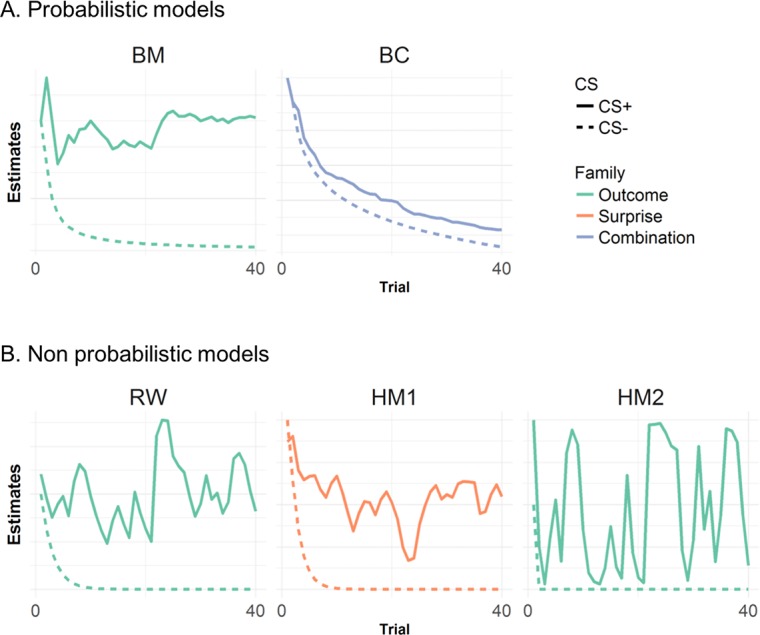
Predictions of probabilistic and non probabilistic RL models, grouped in families according to their predictions. For reasons of consistency with the ANS data displayed on Fig 1, we only show predictions of non-reinforced trials for CS+ (solid lines) and an equal number of CS- trials (equal to 40, dashed lines). (a) Model predictions for probabilistic models. Model predictions are illustrated according to whether they reflect a measure of the estimated CS value (green lines, *outcome*), the degree of surprise/model update (orange lines, *surprise*), or a combination of two quantities (purple lines, *combination*). (b) Model predictions for non probabilistic models, similar to -a-. This figure shows predictions for an exemplar trial sequence, to illustrate the trial-by-trial dynamics of each model. See S1 Fig for averages over 100 trial sequences, illustrating the general trends of the predictions.

To explain trial-by-trial changes in ANS responses, we considered a variety of learning models and different mappings from model onto ANS response (Table 3, Fig 2, S1 Fig). We included a Rescorla-Wagner (RW) model [13] which in our paradigm makes similar predictions as a more general temporal difference rule [32]. A hybrid RW-Pearce-Hall model (HM) [33] which has been used to specifically explain SCR in threat learning, and augments the RW model with a notion of associability [14]. We then considered different observation functions. For the RW model, the only straightforward observation function contains the US expectation. For the hybrid model, the independent variable in the observation function was either associability as previously proposed (HM1) or US expectation (HM2), and for the probabilistic model it was either US expectation (BM) or a combination of US expectation and estimated uncertainty of the environment (BC). As null models, we considered a situation in which no learning occurs over time (i.e. the CS+/CS- difference is time-invariant). We included a probabilistic model that tracks the uncertainty of the environment and does not distinguish CS+/CS- (UN), and a model that distinguishes CS+/CS- but with no change over time (NL). Model evidence was approximated by Bayesian Information Criterion (BIC), which provides a trade-off between model fit and model complexity [34,35].

**Table 3:** Overview of the model space and the independent variables in the observation functions. The last column highlights the type of model quantity that is reflected in the observation function (O = expected outcome, S = surprise/model update, C = Combination of O and U, N = Null).

Model		Independent variable in the observation function	Number of parameters in learning model	Family
**RW**		Associative strength: *x*_*t*_	1	O
**HM**	HM1	Associability: *η*_*t*_	1	S
	HM2	Associative strength: *x*_*t*_	1	O
**BM**		Prior expectation: *E*[*θ*]	0	O
**BC**		Prior uncertainty and prior expectation: *v*_*t*_ + *E*[*θ*]	0	C
**UN**		Prior uncertainty: *v*_*t*_	0	N
**NL**		Constant difference: {1,0}	0	N

### Observation function

We first determined the most likely type of observation function for either autonomic measure, irrespective of the underlying model. To this end, we split models into families, according to whether they reflect quantities that are analogous to the expected outcome (3 models: RW, HM2, BM), a notion of previous surprise (also termed associability [15] model HM1), or a combination of expected outcome and its uncertainty (model BC).

**Fig 3:**
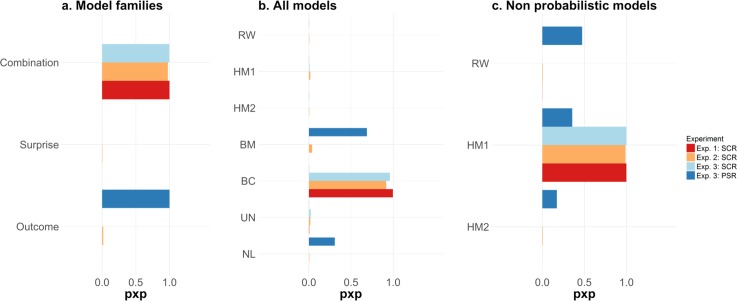
**Model selection results (a) across three families of observation models (b) for the entire model space and (c) for previously proposed non probabilistic models for threat learning.** The three model families of panel (a) collapse across all models and reflect observation functions related to value (models RW, HM2, BM), surprise (model HM1) and combinations (noted Bayesian Combination model, BC). The bars in all panels indicate model evidence, based on protected exceedance probabilities (p.x.p., RFX).

A family-based model comparison confirmed the intuition that SCR and PSR are mapped onto different observation functions (Fig 1): SCR was best explained by a combination of quantities, indexed by protected exceedance probabilities (p.x.p.) of > 0.97 for all three experiments (Fig 3A). On the other hand, PSR was best explained by the expected outcome, (p.x.p. > 0.99, Fig 3A). Within the three models included in the outcome family (RW, HM2 and BM), PSR was best explained by model BM (p.x.p. = 1.00).

### Associative learning models

We subsequently performed a model comparison across the entire model space (7 models in total, Table 3). Within this space, we examined which combination of learning and observation models explained the trial-by-trial trajectory of estimated SCR and PSR. As in the previous comparison, SCR data were best explained by the combination model BC (Fig 3B p.x.p. of ≥ 0.99, 0.92 and 0.96 for Experiments 1–3, respectively). This model implements an ideal observer and maps a mixture of US expectation (prior mean) and uncertainty onto the observation function (Fig 2 for an illustration of model predictions). The latter term—the estimated uncertainty—decreases over time as more instances of the same CS/US contingency are observed (Fig 2 and Table 3). On the other hand, PSR were best explained by model BM, as indexed by p.x.p of 0.69 (Fig 3B). This model implements the same Bayesian observer as BC, but a different observation function, which corresponds to the US expectation alone (Fig 2 and Table 3). Thus, it appears that pupil size unambiguously tracks expected outcome of a CS, unlike SCR.

A crucial difference between previously suggested model HM1 and our model BC is the inclusion of a decay term. This raises a concern that a non-probabilistic model together with a decay term, although not motivated a priori, may explain the data equally well or better. To rule out such a possibility, we asked whether a combination of UN + HM1 would explain the data as well as UN + BM (i.e. BC). We subtracted the estimates of model UN from the SCR data and fitted models BM and HM1 to the residuals. In all three datasets, model BM explained the residuals significantly better than model HM1 (p.x.p. > 0.99), confirming the result that a probabilistic model better accounts for the data than previously suggested ones.

### Model comparison among non-probabilistic models

Next, we sought to relate our results to previous studies that only included non probabilistic models into their analysis [15,16]. Among the RL models previously considered, we found highest evidence for HM1, in which SCR reflect the associability term of the Hybrid RW-PH model (Fig 3C, p.x.p.≥ 0.99, for experiments 1–3). This result is in keeping with previous work [15,16]. For PSR, the highest evidence was obtained for model RW, which reflects the estimated CS values (p.x.p. of 0.47, Fig 3C). While it appears that in our and previous studies HM1 best explains SCR among non probabilistic models, a probabilistic model explained our data decisively better, as highlighted above.

### Accuracy and complexity

**Fig 4:**
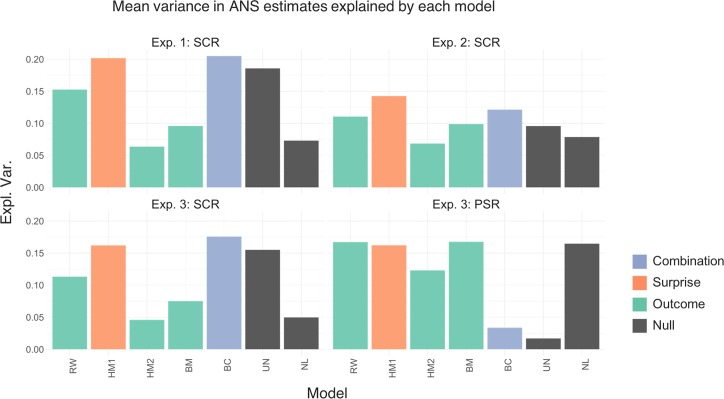
Proportion of variance in the physiological data explained by each of the models across the four experiments. Bar illustrate the explained variance for each model and experiment.

Notably, our probabilistic model is normative and thus contains free parameters only in the observation function, in contrast to all other models. There is a possibility that the least complex model wins in the quantitative model comparison but does not fit the data very well. To rule out this concern, we computed the variance explained by each model, which provides a quantification of the model fit irrespective of its complexity. For SCR, the highest explained variance across participants was obtained for models HM1 and BC (Fig 4): BC won in experiments 1 and 3 (21% and 18% explained variance) and HM1 won in experiment 2. For PSR, BM and RW explained most variance (both 17%). These results suggest that the probabilistic model fits the data equally well as previously proposed models but with fewer parameters.

#### Simulated model recovery results

Finally, we conducted simulations to determine the *a priori* ability of our experimental design to discriminate between the candidate models [36]. We based these simulations on a previously published independent dataset [37]with the same experimental design as the current experiments. From this independent dataset, we derived prior information on the distribution of model parameters. Using these distributions, we simulated data for each model in this study, performed model selection across the whole model space, and determined if the true model was selected. This procedure allowed us to estimate the *a priori* probability of recovering the true model.

**Fig 5:**
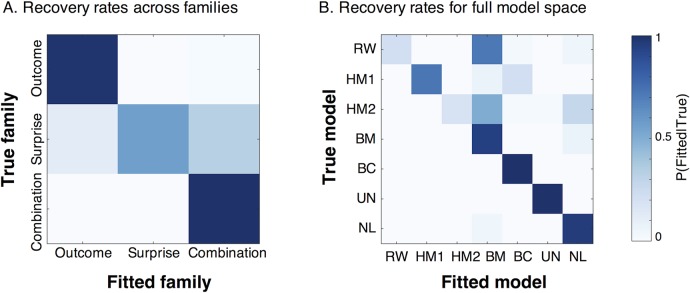
Confusion matrices for family and model recovery rates, using BIC as the evidence metric. (a) Recovery rates across families. Rows represent true models/families (used to simulate data), and columns represent fitted models/families. (b) Recovery rates for single models, similar to -a-. Results are based on 256 simulated experiments, each with 20 participants.

Results of this analysis showed that the probability of recovering the true model family, and the true model, are on average 0.85 and 0.71 respectively (Fig 5). Importantly, the probability of recovering the models that best fit our current data is above 0.94 (0.94 for BM and 1.00 for BC).

## Discussion

Computational understanding of Pavlovian threat learning in biological organisms is limited. In particular, there is no conclusive evidence whether the problem of learning threat probabilities in a structured environment can be solved in the same way as has been proposed for reward learning. In the current study, we compared predictions of associative learning models to the trial-by-trial trajectories of human ANS responses across three data sets and two different ANS readouts. We show that a normative probabilistic model provides a more parsimonious explanation than previously proposed RL models for threat learning for all experiments and both ANS readouts. SCR and PSR are best described by different observation functions, independent of the underlying learning model: PSR can be best explained by estimated outcome prediction, and SCR by a mixture of estimated outcome prediction and its associated uncertainty which is decreasing over time in our task.

The best fitting model for SCR explicitly maps a notion of uncertainty onto trial-by-trial SCR estimates. While such a notion can be built into non probabilistic models [18] it is integral to probabilistic ones. Posterior model checks show that particularly for SCR, the notion of reduced uncertainty over the course of the experiment explains a crucial data feature, namely habituation for both CS+ and CS- trials, a phenomenon that we have previously shown is likely to occur in the central nervous system rather than in the effector organ [29]. A crucial difference between previously proposed RL and our probabilistic model is that in the probabilistic model, effective learning rate is variable over trials, and governed by uncertainty on the prediction while it is fixed in the other models. This probabilistic model assumes that the agent is fully informed about task structure, transition probabilities are stationary, and the US occurrence is governed by a Bernoulli process, i.e., independent between trials. The assumption of stationary outcome probabilities may be appropriate in biological contexts where the predictability of environmental cues does not change over time but between contexts. However, there is evidence that human participants can update slowly changing threat probabilities, as in the case of gradual extinction [18], which is appropriate under a small degree of environmental uncertainty, which may be modelled in hierarchical models [19,38]. It will require more sophisticated trial sequences to determine which uncertainty assumptions are implemented in the human threat learning system. For the moment, one may conclude that the flexibility or statistical complexity engendered by non-probabilistic models does not offer a better explanation of ANS data.

Since probabilistic terms can be incorporated into RL models [18], it is possible that the neural circuits implementing threat learning use a RL model with these features. However, while there is ample neural evidence for neural representation of prediction errors during reward conditioning [39–43], evidence on the nature of prediction error signals for threat learning is contradictory [44–46]. Notably, probabilistic models explain other neural systems better than non probabilistic RL models, for example in explaining putative prediction error signals during sensory perception of auditory regularities [47], or in modelling choice-based correlates of associative learning [21,48], in line with other sources of evidence for probabilistic neural computations [49]

### Quantification of learning

While humans may show overt behaviour during Pavlovian fear conditioning, such as freezing [50], formal attempts to study such phenomena in humans remain limited [51]. This is why we relied on autonomic readouts, the most common way of quantifying fear in humans [2]. In order to analyse ANS activity, we employed a statistical framework for modelling outputs of the peripheral nervous system, such as the SCR and PSR [28,52]. In this framework, estimates of ANS impulse amplitude are obtained using independently validated canonical functions that map ANS firing bursts to peripheral output, with a priori defined constraints on ANS burst timing. Presumably, these estimates more directly reflect central neural states than the noisy peripheral responses themselves. Indeed, ANS amplitude estimates derived from this approach have been shown to better distinguish CS+ and CS- than other approaches, not only for SCR [30,52] and PSR [31] but also for fear-conditioned bradycardia [53], respiratory amplitude responses [54] and fear-potentiated startle [55]. Whether these latter readouts reflect similar quantities as SCR and PSR, and are controlled by the same learning algorithm, remains a topic for future investigation.

### Comparison with previous work

Our results extend previous fear conditioning studies which were restricted to Rescorla-Wagner and hybrid Rescorla-Wagner-Pearce-Hall models [15,16,56]. Importantly, these studies demonstrated that changes in trial-by-trial SCR can be better explained by an associability term of a RW-PH hybrid model than the US prediction itself [15,16]. When considering non probabilistic RL models only, we could replicate these results, showing that our approach is indeed sensitive and able to capture the same learning mechanisms as previously reported. By using more precise trial-by-trial ANS response estimates and including a probabilistic model, we highlight a different underlying learning mechanism, but conceptually confirm that SCR do not reflect US predictions. In our winning model, the observation function is a combination of the expected US (prior mean) and estimated uncertainty, which depends on the total number of observations during the experiment. Interestingly, there is qualitative evidence that PSR may be informative of past prediction errors [57], something that we did not confirm here. More elaborate experimental designs with specific trial sequences may be required to shed light on this question.

### Limitations

Our probabilistic model is normative under stationary outcome probabilities and allows incorporating prior information in the form of beta distributions. This formalism is mathematically elegant but even under an in-built assumption of stationarity it may appear too specific as a candidate for a neural model. Furthermore, there is weak evidence towards a decaying CS+/CS- difference over time, something that requires future experiments to be determined and that none of the models considered here could predict. While our probabilistic model makes the strong assumption of a logarithmic decay of SCR over time, this prediction was not quantitatively corroborated in the current data set. We note that Bayesian models with more generic distributional assumptions such as a Kalman temporal difference filter [18] or an hierarchical Gaussian filter [19] make very similar predictions under the experimental circumstances employed here, such that more sophisticated procedures will be required to disentangle different Bayesian models. Future work may thus elucidate plausible neural implementations that predict the progression of ANS responses highlighted here, which may be distinguished by hemodynamic or electrophysiological activity to formalise the neural implementation of aversive learning.

## Materials and methods

### Ethics statement

The study was performed in accordance with the Declaration of Helsinki, and all procedures for all experiments were approved by the governmental ethics committee (Kantonale Ethikkomission Zurich). All participants gave written informed consent using a form approved by the ethics committee.

### Design & Participants

We conducted three fear conditioning experiments using delay and trace conditioning, and visual as well as auditory CS. A fourth control experiment was used for optimising SCR analysis. Four independent samples of participants (overall N = 102) were recruited from the general and student population (see Table 1 for a detailed sample description).

### Experimental procedure

#### Experiment 1

Experiment 1 was a standard discriminant delay conditioning task, in which participants were presented with two neutral CS, consisting of a blue or red background screen presented for 4 s. One CS co-terminated on 50% of trials with an unconditioned stimulus (US); the other was never reinforced. US was an aversive 500 ms train of electrical square pulses (500 Hz, 50% duty cycle), delivered to participants’ right forearm through a pin-cathode/ring-anode arrangement. Intensity was set to be clearly discomforting in a two-step procedure: first, we gradually increased the US intensity, up to a level that was perceived by participants as painful. Second, we delivered to participants 14 stimulations of random intensity, never exceeding the pain threshold, identified in the first step. Participants were asked to rate each stimulation on a scale of 0–100 (i.e. not perceived at all to clearly painful). For the actual experiment, we delivered the intensity that participants reported as corresponding to 85% of this scale, and confirmed that it corresponded to a discomforting, but not painful level.

During an inter-trial interval randomly determined to last 7, 9 or 11 s, a black background screen was shown. Participants were presented with 80 CS+ and 80 CS- trials split in two blocks. The first trial of each block was always a reinforced CS+, while the order of the remaining trials within each block was randomized for each participant separately.

To keep participants alert, they were instructed to indicate through a button press the colour they saw on the monitor. The CS colours and colour-button associations were counter-balanced across participants. Participants were instructed that "one CS may be more likely to be followed by an electric shock than the other".

#### Experiment 2

Experiment 2 was a standard discriminant trace conditioning paradigm, with the same type of CS and US as experiment 1. CS were presented for 3 s, followed by a 1 s trace interval during which a black screen appeared. US were delivered after the trace interval, in 50% of the CS+ trials.

#### Experiment 3

Experiment 3 was a delay fear conditioning paradigm, using similar settings as experiment 1, but with auditory CS to allow measuring pupil responses independent of luminance influences [28]. CS were two sine tones with constant frequency (220 or 440 Hz), approximate loudness of 60 dB, lasting 4 s and delivered via headphones (HD 518, Sennheiser, Wendemark-Wennebostel, Germany). Participants were asked to fixate on a white cross (height/width 1.67° visual angle), presented on a gray background (72.7 cd/m^2^).

### Control experiment

The fourth experiment was identical to experiment 1, and was carried out in a separate group of participants, in order to optimise the estimation of anticipatory sympathetic arousal in an unbiased way.

### Data recording

All experiments took place in a dark, soundproof chamber with background illumination provided by the camera and the monitor lights. Participants’ heads were positioned on a chin rest with 70 cm distance from the monitor (Dell P2012H, 20” set to an aspect ratio of 5:4, 60 Hz refresh rate). Skin conductance was recorded from the thenar/hypothenar of participants' left hand, using 8 mm Ag/AgCl cup electrodes (EL258, Biopac Systems Inc., Goleta, CA, US) and 0.5% NaCl gel (GEL101, Biopac) [58]. Skin conductance signal was amplified with an SCR coupler/amplifier (V71-23, Coulbourn Instruments, Coulbourn Instruments, Whitehall, PA, US). Data were digitised at 1000 Hz (DI-149, Dataq Instruments, Akron, OH, US), and recorded with Windaq (Dataq Instruments) software. In experiment 3, an EyeLink 1000 System (SR Research, Ottawa, Ontario, Canada) was used for recording pupil diameter and gaze direction with a sampling rate of 500 Hz. Before the experiment we calibrated gaze direction using a 9-point calibration procedure, implemented in the EyeLink 1000 software.

### Data pre-processing and analysis

All physiological data were analysed using PsPM, version 3.0.2 (http://pspm.sourceforge.net).

### Skin conductance

SCR data were filtered offline with band-pass bidirectional Butterworth filter (cut-off frequencies 0.0159–5 Hz) and then down-sampled to 10 Hz. We visually inspected the averaged SCR to the US, which normally starts 1–2 s after US onset [59]. Since fear conditioning requires an aversive US, participants who did not show such a US response (defined as a positive peak on the averaged SCR responses exceeding 0.05 μS relative to a baseline of 2 s before CS onset) were conservatively excluded. This removed 7 participants from experiment 1 and 1 participant from experiment 2.

Skin conductance was analysed with a non-linear model (dynamic causal model, DCM) of the anticipatory SCR (aSCR). Note that this approach has no conceptual similarity to the connectivity DCMs used in neuroimaging but uses the same statistical machinery for inversion. This procedure infers activity of the sympathetic nervous system, given changes in the recorded SCR signal [59] and provides trial-by-trial estimates of anticipatory sympathetic responses. For this analysis, we used the default DCM pre-processing and inversion, as implemented in PsPM 3.0.2 [30,59]. Timing parameters of the neural input model were initially optimised by using data from an independent experiment, as described below.

### Optimisation of SCR analysis

Prior to all SCR analyses, we optimised the psychophysiological model (PsPM) which specifies the timing of sudomotor inputs into the skin/sweat-gland system with respect to CS. To this end, we used data from a fourth, control experiment, which was not further used for the reinforcement modelling part of the study; hence there is no risk of circularity.

Our PsPM describes how sympathetic arousal generates sudomotor impulses, which in turn generate SCR. This model is inverted to yield estimates of sudomotor impulse amplitude. This reduces the influence of non-specific SCR (e. g. spontaneous fluctuations) which are unrelated to the experiment and may overlap with experiment-induced SCR. Because the timing of sudomotor impulses is not known a priori, there are different plausible ways to specify a PsPM; each offers a slightly different balance between model realism and influence of SCR unrelated to the experiment, and thus, a possibly different signal-to-noise ratio. To empirically determine the best PsPM specification for the current experiment, we compared various specifications in their ability to distinguish between CS+ and CS-, something we have previously termed predictive validity [26].

In previous publications we defined a window of sudomotor impulses from CS onset to CS offset and loosely constrained the temporal dispersion of these impulses. This is the most unconstrained definition and based only on the fact that anticipatory arousal must occur after the CS identity is known by the observer, and before the outcome occurs [5,59]. Here, we investigated whether constraining the anticipatory time window improved predictive validity. We fixed the onset of the anticipatory window at CS onset and varied its duration from 0 to 3.5 s in steps of 0.5 s. For all models, we considered an additional component reflecting US responses, with fixed onset at 3.5 s (i.e. US delivery or omission).

For each of the resulting models we considered the trial type (CS+/CS-) as dependent variable in a linear regression model, while the estimated responses for each participant, together with participant-specific intercepts were considered as predictors. The F-statistic of this model is identical to the squared t-value from a paired t-test. The Residual Sum of Squares (RSS) of this regression was then converted to Bayesian information criterion (BIC) [60]:

$$BIC=n\cdot \log\;\left(\frac{1}{n}RSS\right)+c$$

Where n is the number of observations and c a complexity constant that is the same for all models.

This comparison showed that the model with the lowest BIC value consisted of a fixed component at the onset of the CS with an offset of 0 s (BIC = -76.74). The BIC of the remaining models ranged from -73.29 to -59.19. Therefore, for all subsequent analyses of SCR data, we implemented a DCM with a fixed latency response at CS onset and a fixed latency response at US onset or omission, for each trial.

### Pupil

Time-series of pupil size data were extracted from the EyeLink 1000 System, after online parsing for saccade and fixation losses with an in-built algorithm. All data points during eye blinks, head movements, or periods where gaze positions along the x or y axis deviated more than ± 5° visual angle from the fixation, were removed and linearly interpolated [28]. For each participant, we analyzed either the left or right pupil, for whichever there were more data points available. We then estimated the anticipatory input into the pupil with single-trial general linear convolution models (GLMs). Each regressor in the model was formed by convolving a stick function at CS onset for one trial with a synthetic pupil response function previously developed [28,31]. The GLM included one regressor per single trial.

### Statistical contrast of SCR / PSR estimates

We statistically contrasted physiological estimates across trials and participants using linear mixed effects models (LME, package nlme) in R (www.r-project.org; version 3.2). We considered CS (CS+/-) and Trial as fixed factors and Participant as random, to account for inter-participant variance:

ANS˜1+CS*Trial,random=˜1|Participant.

LME results were Bonferroni-corrected to account for multiple comparisons across the tested datasets.

### Behavioural models

We assumed that on every trial *t*, ANS responses, *y*_*t*_, are a linear function of the output of an associative learning model plus a noise term, *ϵ*_*t*_:

$$y_{t}=\beta_{1}\cdot z_{t}+\beta_{0}+\epsilon_{t},\;\epsilon_{t}∼N(0,\sigma_{\epsilon }^{2}),$$

where *z*_*t*_ is a model variable that links a learning model to the ANS output, while *β*_1_ and *β*_0_ are model- and participant-specific free parameters. The linear mapping between model output and ANS response is motivated by simplicity, and by the previous observations that in fear conditioning, US probability linear maps onto SCR [61], and that SCR is linearly related to the underlying neural response [62].

### Probabilistic models

We used a Bayesian learning model, which assumes a Bayesian agent that represents threat predictions probabilistically [63,64]. Specifically, a model of the discrete US prediction (Bernoulli probability) with parameter *θ*_*CS*_ for each of the two CS is sequentially updated based on new sensory information and prior beliefs, according to Bayes’ rule:

$$p_{t}(\theta |u_{t})=\frac{p(u_{t}|\theta )\cdot p_{t-1}(\theta |u_{t-1})}{p(u_{t})}$$

where *u*_*t*_ is the presence or absence of US input at trial *t*.

In our case, p(u_t_ | *θ*), the likelihood function for each individual trial, follows a Bernoulli distribution, as trials can have two possible outcomes (US or no US):

$$p(u_{t}|\theta )=\theta^{u_{t}}{\left(1-\theta \right)}^{1-u_{t}},$$

where *u*_*t*_ ∈ {0,1}. The prior probability distribution before the first trial, *p*_0_(*θ* | *u*_*t*−1_) was assumed to follow a Beta distribution, which is conjugate to the Bernouilli, and which has a probability density function:

$$p(\theta |u_{t-1})=\frac{\theta^{\alpha_{t-1}}{\left(1-\theta \right)}^{\beta_{t-1}}}{Β(\alpha_{t},\beta_{t})},$$

where B(*α*_*t*_,*β*_*t*_) is a Beta function with parameters *α*_*t*_ and *β*_*t*_.

The resulting posterior distribution is thus also a Beta distribution, whose parameters are updated according to: *α*_*t*_ = *α*_*t*−1_ + *u*_*t*−1_ and *β*_*t*_ = *β*_*t*−1_ − *u*_*t*−1_ + 1, where *u*_*t*_ = 1 if a US occurred, and *u*_*t*_ = 0 otherwise. We assumed uninformative initial priors for the Bayesian model with *α*_0_ = *β*_0_ = 1, such that the model essentially reflects an ideal Bayesian learner.

Although other probabilistic models with more realistic distributional assumptions can account for learning in a Bayesian framework [18,65], they will generate very similar predictions when the prior distribution is uninformative. Hence, we used the Beta-Binomial model for the sake of simplicity: the resulting conjugate Beta posterior distribution has a support in (0, 1) and can readily account for the probability of a US to occur.

This model allows for different observation functions (Table 3). We considered (a) the mean of the prior distribution (BM), and (b) a combination of (a) with the uncertainty of the environment (BC).

a. Prior mean (BM)

This response function maps *E*[*θ*], the mean of the prior distribution, onto ANS responses:

$$E[\theta ]=\int_{\theta }^{}\theta \cdot p(\theta |u_{t-1})d\theta .$$

For the case of a beta distribution the model output, mapped on the independent variable *z*_*t*_, corresponds to:

$$z_{t}=E[\theta ]=\frac{\alpha_{t-1}}{\alpha_{t-1}+\beta_{t-1}}.$$

b. Combined prior mean and prior uncertainty model (BC)

The logarithm of total amount of observations drawn by participants has been previously used to quantify the uncertainty in a changing environment [38]. In our case, where the environment is static and contingencies do not change over the time-course of the experiment, this term is related to the prior uncertainty, or in other words, to the sharpness of the prior distribution.

$$v_{t}=-\ln\left(\alpha_{t-1}+\beta_{t-1}\right).$$

This quantity was computed for each CS separately. A wide distribution corresponds to large values of *v*_*t*_ and would allow for large changes in the mean of this distribution from one trial to the next [38].

We considered a model whose independent variable, *z*_*t*_, comprises a mixture of the prior mean and uncertainty:

$$z_{t}=v_{t}+E[\theta ].$$

### Previously proposed non probabilistic models

We included into our analysis a range of classical, non-probabilistic reinforcement learning models in which point estimates of US predictions are updated according to some learning rule that involves signed or unsigned prediction errors.

a. Rescorla-Wagner rule (RW)

The RW model [13] specifies that US prediction ('associative strength') is updated according to a signed prediction error signal (i.e. the difference between the prediction on a given trial, *x*_*t*_ and the observed sensory input, *u*_*t*_), weighted by a fixed learning rate, *η* which is a subject-specific free parameter across both CS, such that such that 0<*η*<1:

$$x_{t}=x_{t-1}+\eta \cdot (u_{t-1}-x_{t-1}).$$

In our case, where there is only one type of US, which is either present or absent, *u*_*t*_ can take two values: *u*_*t*_ = 1, if a US was presented on a given trial and *u*_*t*_ = 0 otherwise. We assumed that participants had no specific expectations about the two stimuli and thus set the starting values *x*_0_ = 0.5 for both CS+/CS-. For the RW model we used only one observation function (Table 3):

$$z_{t}=x_{t}.$$

b. Hybrid RW/PH model (HM)

The Rescorla-Wagner and Pearce-Hall models can be combined into a hybrid model (HM), which assumes signed prediction errors (like a RW rule), and an extra associability term, *η*_*t*_ [15,16,66]. This quantity, *η*_*t*_, reflects a dynamic learning rate, which is updated over the course of the experiment according to:

$$\eta_{t}=k\cdot |x_{t-1}-u_{t-1}|+(1-k)\cdot \eta_{t-1},$$

$$x_{t}=x_{t-1}+\eta_{t-1}\cdot (u_{t-1}-x_{t-1}),$$

where k is a participant-specific positive free scaling parameter common to both CS, reflecting how fast each participant’s predictions are updated, with 0<k<1. As for the RW model, we assumed that participants had no specific expectations about the two stimuli and thus set the initial values at *x*_0_ = 0.5 for both CS+/CS-.

HM could have two different observation functions to ANS responses: the current associability (HM1), and the current US prediction (HM2) Hence, the two possible observation functions of the hybrid model are (Table 3):

$$z_{t}=\eta_{t},$$

$$z_{t}=x_{t}.$$

### Null models

We additionally considered two null models. The first of them, model VO, only reflects the total amount of observations drawn by participants:

$$z_{t}=-\ln\left(\alpha_{t}+\beta_{t}\right).$$

This model could explain effects of habituation, in the absence of any learning.

Finally, we included a null model, which assumes that no habituation takes place over the course of the experiment, and that learning takes place prior to the experiment (i.e. the CS+/CS- difference is constant over time):

$$z_{t}=\{1,0\}.$$

### Model fit and selection

Models were optimised for each participant separately, by minimising the residual sum of squares (RSS) between each model’s predictions and trial-by-trial estimates of anticipatory neural responses:

$$RSS_{MM}=\sum_{t=1}^{T}{\left(\beta_{1}\cdot z_{t}+\beta_{0}-y_{t}\right)}^{2},$$

where *T* is the total number of non-reinforced CS+ and CS- for each experiment. All trials were used for generating model predictions, but only trials which were not paired with a US were taken into account when computing the RSS, in order to avoid contamination by an evoked response to the US, an approach similar to previous studies [15]. The free parameters of each model and two regression parameters of the observation function were fitted using an interior point search algorithm as implemented in the Matlab function fmincon, using the RSS as objective function.

Model evidence was approximated by Bayesian Information Criterion (BIC), which was calculated for each model and participant [34,35]:

$$BIC_{MM}=p\cdot ln(T)+T\cdot ln\frac{RSS_{MM}}{T},$$

where *p* refers to the total number of parameters for each model and T to the total number of observations (i.e. single trials unpaired with a US). BIC approximates the true model evidence and provides a compromise between model fit and model complexity, indicated by the total number of free parameters in each model, *p*. For all models, *p* ≥ 2, since at least two parameters were estimated for the linear mapping between transfer functions, *g*, and the psychophysiological data.

Model selection was performed at the group level, using random effects analysis (RFX), [67,68], which treats models as random effects in the population. RFX was based on protected exceedance probabilities (p.x.p.), which quantify the probability of a model to be more likely than any other, give the group data, using SMP12 (http://www.fil.ion.ucl.ac.uk/spm/software/spm/). RFX originally accounts for the possibility that different individuals use different models. This is unlikely to be the case in an evolutionarily conserved mechanism like threat learning, but RFX also provides good protection against outliers which is why we used it here.

### Simulated model recovery

To examine the *a priori* model selection error of the given experimental design and model space, we simulated data based on an already published dataset with an independent and larger sample of participants in the same experimental design [37]. We fitted all of our models to skin conductance data obtained during the acquisition phase from the placebo participant group (N = 38) of this study. This yielded, for each model, an empirical distribution of participant-wise parameters. Then, we simulated 256 experiments with 20 participants each (i.e. a sample size similar to that of the individual experiments in the manuscript), by bootstrapping from the estimated distribution of parameter values. To the simulated datasets we fitted all the models and computed model recovery rates using BIC as the model selection criterion.

## Supporting information

S1 Fig Mean model estimates, over 100 simulated CS+/- and reinforcement sequences. (PDF)

S2 Fig Results of LME fitting for SCR and PSR for the three experiments. (PDF)

## References

[1] LeDoux JE. Coming to terms with fear. Proc Natl Acad Sci U S A. 2014;111: 2871–8. 10.1073/pnas.1400335111 24501122 PMC3939902

[2] LeDoux JE. Emotion circuits in the brain. Annu Rev Neurosci. 2000;23: 155–184. 10.1146/annurev.neuro.23.1.155 10845062

[3] Ciocchi S, Herry C, Grenier F, Wolff SBE, Letzkus JJ, Vlachos I, et al Encoding of conditioned fear in central amygdala inhibitory circuits. Nature. 2010;468: 277–282. 10.1038/nature09559 21068837

[4] McNally GP, Johansen JP, Blair HT. Placing prediction into the fear circuit. Trends Neurosci. Elsevier Ltd; 2011;34: 283–292. 10.1016/j.tins.2011.03.005 21549434 PMC4245078

[5] Bach DR, Weiskopf N, Dolan RJ. A Stable Sparse Fear Memory Trace in Human Amygdala. J Neurosci. 2011;31: 9383–9389. 10.1523/JNEUROSCI.1524-11.2011 21697388 PMC3146048

[6] Schultz W, Dayan P, Montague PR. A Neural Substrate of Prediction and Reward. Science (80-). 1997;275: 1593–1599. 10.1126/science.275.5306.1593 9054347

[7] Kim EJ, Horovitz O, Pellman B a, Tan LM, Li Q, Richter-Levin G, et al Dorsal periaqueductal gray-amygdala pathway conveys both innate and learned fear responses in rats. Proc Natl Acad Sci U S A. 2013;110: 14795–800. 10.1073/pnas.1310845110 23959880 PMC3767534

[8] Letzkus JJ, Wolff SBE, Meyer EMM, Tovote P, Courtin J, Herry C, et al A disinhibitory microcircuit for associative fear learning in the auditory cortex. Nature. 2011;480: 331–335. 10.1038/nature10674 22158104

[9] Krasne FB, Fanselow MS, Zelikowsky M. Design of a neurally plausible model of fear learning. Front Behav Neurosci. 2011;5: 41 10.3389/fnbeh.2011.00041 21845175 PMC3145244

[10] Madarasz TJ, Diaz-Mataix L, Akhand O, Ycu EA, LeDoux JE, Johansen JP. Evaluation of ambiguous associations in the amygdala by learning the structure of the environment. Nat Neurosci. 2016;19: 965–972. 10.1038/nn.4308 27214568 PMC5655997

[11] Mackintosh NJ. Conditioning and associative learning. Oxford Psychology Series; 1983 10.1016/0005-7967(85)90036-1

[12] Gershman SJ, Monfils MH, Norman KA, Niv Y. The computational nature of memory modification. Elife. 2017;6 10.7554/eLife.23763 28294944 PMC5391211

[13] Rescorla R a, Wagner a R. A theory of Pavlovian conditioning: Variations in the effectiveness of reinforcement and nonreinforcement. Class Cond II Curr Res Theory. 1972;21: 64–99. 10.1101/gr.110528.110

[14] Pearce JM, Hall G. A model for Pavlovian learning: variations in the effectiveness of conditioned but not of unconditioned stimuli. Psychol Rev. 1980;87: 532–552. 10.1037/0033-295X.87.6.532 7443916

[15] Zhang S, Mano H, Ganesh G, Robbins T, Seymour B. Dissociable Learning Processes Underlie Human Pain Conditioning. Curr Biol. The Authors; 2015; 1–7. 10.1016/j.cub.2015.10.066 26711494 PMC4712170

[16] Li J, Schiller D, Schoenbaum G, Phelps EA, Daw ND. Differential roles of human striatum and amygdala in associative learning. Nat Neurosci. Nature Publishing Group; 2011;14: 1250–1252. 10.1038/nn.2904 21909088 PMC3268261

[17] Dayan P, Abbott LF. Theoretical Neuroscience. 2005.

[18] Gershman SJ. A Unifying Probabilistic View of Associative Learning. PLoS Comput Biol. 2015;11: 1–20. 10.1371/journal.pcbi.1004567 26535896 PMC4633133

[19] Mathys C, Daunizeau J, Friston KJ, Stephan KE. A bayesian foundation for individual learning under uncertainty. Front Hum Neurosci. 2011;5: 39 10.3389/fnhum.2011.00039 21629826 PMC3096853

[20] Bach DR, Dayan P. Algorithms for survival: A comparative perspective on emotions. Nat Rev Neurosci. 2017;18: 311–319. 10.1038/nrn.2017.35 28360419

[21] Stankevicius A, Huys QJM, Kalra A, Seriès P. Optimism as a Prior Belief about the Probability of Future Reward. PLoS Comput Biol. 2014;10 10.1371/journal.pcbi.1003605 24853098 PMC4031045

[22] Boucsein W. Electrodermal activity. 2012.

[23] McDougal D, Gamlin P. Pupillary Control Pathways In: Basbaum A, Kaneko A, editors. The senses: A comprehensive reference. San Diego, CA: Academic Press; 2008 pp. 521–536.

[24] Wood KH, Ver Hoef LW, Knight DC. The amygdala mediates the emotional modulation of threat-elicited skin conductance response. Emotion. 2014;14: 693–700. 10.1037/a0036636 24866521 PMC4115032

[25] Koikegami H, Yoshida K. Pupillary Dilatation Induced By Stimulation of Amygdaloid Nuclei. Psychiatry Clin Neurosci. 1953;7: 109–126. 10.1111/j.1440-1819.1953.tb00600.x 13142113

[26] Bach DR, Friston KJ. Model-based analysis of skin conductance responses: Towards causal models in psychophysiology. Psychophysiology. 2013;50: 15–22. 10.1111/j.1469-8986.2012.01483.x 23094650

[27] Bach DR, Flandin G, Friston KJ, Dolan RJ. Time-series analysis for rapid event-related skin conductance responses. J Neurosci Methods. 2009;184: 224–234. 10.1016/j.jneumeth.2009.08.005 19686778 PMC2772899

[28] Korn CW, Bach DR. A solid frame for the window on cognition: Modeling event- related pupil responses. 2016;16: 1–16. 10.1167/16.3.28 26894512 PMC4993241

[29] Gerster S, Namer B, Elam M, Bach DR. Testing a linear time invariant model for skin conductance responses by intraneural recording and stimulation. Psychophysiology. 2017; 10.1111/psyp.12986 28862764 PMC5811801

[30] Staib M, Castegnetti G, Bach DR. Optimising a model-based approach to inferring fear learning from skin conductance responses. J Neurosci Methods. Elsevier B.V.; 2015;255: 131–138. 10.1016/j.jneumeth.2015.08.009 26291885 PMC4612446

[31] Korn CW, Staib M, Tzovara A, Castegnetti G, Bach DR. Pupil size response model indexes fear conditioning. Under Revis. 2016; 10.1111/psyp.12801 PMC5324687 27925650

[32] O’Doherty JP, Dayan P, Friston K, Critchley H, Dolan RJ. Temporal difference models and reward-related learning in the human brain RID D-9230-2011. Neuron. 2003;38: 329–337. 10.1016/S0896-6273(03)00169-7 12718865

[33] Li W, Howard JD, Parrish TB, Gottfried J a. Aversive learning enhances perceptual and cortical discrimination of indiscriminable odor cues. Science. 2008;319: 1842–1845. 10.1126/science.1152837 18369149 PMC2756335

[34] Raftery AE. Bayesian model selection in social research. Sociol Methodol. 1995;25: 111–163.

[35] Penny WD, Stephan KE, Mechelli A, Friston KJ. Comparing dynamic causal models. Neuroimage. 2004;22: 1157–1172. 10.1016/j.neuroimage.2004.03.026 15219588

[36] Palminteri S, Wyart V, Koechlin E. The Importance of Falsification in Computational Cognitive Modeling. Trends Cogn Sci. 2017;21: 425–433. 10.1016/j.tics.2017.03.011 28476348

[37] Bach DR, Tzovara A, Vunder J. Blocking human fear memory with the matrix metalloproteinase inhibitor doxycycline. Mol Psychiatry. 2017; 10.1038/mp.2017.65 28373691 PMC5507298

[38] Behrens TEJ, Woolrich MW, Walton ME, Rushworth MFS. Learning the value of information in an uncertain world. Nat Neurosci. 2007;10: 1214–21. 10.1038/nn1954 17676057

[39] Montague PR, Dayan P, Sejnowski TJ. A framework for mesencephalic dopamine systems based on predictive Hebbian learning. J Neurosci. 1996;16: 1936–1947. doi: 10.1.1.156.635 8774460 10.1523/JNEUROSCI.16-05-01936.1996 PMC6578666

[40] Schultz W, Dayan P, Montague PR. A neural substrate of prediction and reward. Science (80-). 1997;275: 1593–1599. 10.1126/science.275.5306.1593 9054347

[41] Bayer HM, Glimcher PW. Midbrain dopamine neurons encode a quantitative reward prediction error signal. Neuron. 2005;47: 129–141. 10.1016/j.neuron.2005.05.020 15996553 PMC1564381

[42] Bryden DW, Johnson EE, Tobia SC, Kashtelyan V, Roesch MR. Attention for learning signals in anterior cingulate cortex. J Neurosci. 2011;31: 18266–18274. 10.1523/JNEUROSCI.4715-11.2011 22171031 PMC3285822

[43] Klavir O, Genud-Gabai R, Paz R. Functional connectivity between amygdala and cingulate cortex for adaptive aversive learning. Neuron. Elsevier Inc.; 2013;80: 1290–1300. 10.1016/j.neuron.2013.09.035 24314732

[44] Schultz W, Wise R, Rompre P-P, Everitt B, Robbins T, Bernoulli D, et al Dopamine signals for reward value and risk: basic and recent data. Behav Brain Funct. 2010;6: 24 10.1186/1744-9081-6-24 20416052 PMC2876988

[45] Cohen JY, Haesler S, Vong L, Lowell BB, Uchida N. Neuron-type-specific signals for reward and punishment in the ventral tegmental area. Nature. 2012;482: 85–88. 10.1038/nature10754 22258508 PMC3271183

[46] McHugh SB, Barkus C, Huber A, Capitão L, Lima J, Lowry JP, et al Aversive prediction error signals in the amygdala. J Neurosci. 2014;34: 9024–33. 10.1523/JNEUROSCI.4465-13.2014 24990922 PMC4078079

[47] Lieder F, Daunizeau J, Garrido MI, Friston KJ, Stephan KE. Modelling Trial-by-Trial Changes in the Mismatch Negativity. PLoS Comput Biol. 2013;9 10.1371/journal.pcbi.1002911 23436989 PMC3578779

[48] Costa VD, Tran VL, Turchi J, Averbeck BB. Reversal learning and dopamine: a bayesian perspective. J Neurosci. 2015;35: 2407–16. 10.1523/JNEUROSCI.1989-14.2015 25673835 PMC4323525

[49] Bach DR, Dolan RJ. Knowing how much you don’t know: A neural organization of uncertainty estimates. Nat Rev Neurosci. 2012;13: 572–586. 10.1038/nrn3289 22781958

[50] LeDoux J, Ciocchetti P, Xagoraris A, Romanski L. The lateral amygdaloid nucleus: sensory interface of the amygdala in fear conditioning. J Neurosci. 1990;10(4): 1062–1069. 2329367 2329367 10.1523/JNEUROSCI.10-04-01062.1990 PMC6570227

[51] Roelofs K. Freeze for action: neurobiological mechanisms in animal and human freezing. 2017; 10.1098/rstb.2016.0206 28242739 PMC5332864

[52] Bach DR, Flandin G, Friston KJ, Dolan RJ. Modelling event-related skin conductance responses. Int J Psychophysiol. 2010;75: 349–356. 10.1016/j.ijpsycho.2010.01.005 20093150 PMC2877881

[53] Castegnetti G, Tzovara A, Staib M, Paulus PC, Hofer N, Bach DR. Modeling fear-conditioned bradycardia in humans. Psychophysiology. 2016;53: 930–939. 10.1111/psyp.12637 26950648 PMC4869680

[54] Castegnetti G, Tzovara A, Staib M, Gerster S, Bach DR. Assessing fear learning via conditioned respiratory amplitude responses. Psychophysiology. 2016; 10.1111/PSYP.12778 27933608 PMC6001548

[55] Khemka S, Tzovara A, Gerster S, Quednow BB, Bach DR. Modeling startle eyeblink electromyogram to assess fear learning. Psychophysiology. 2016; 10.1111/psyp.12775 27753123 PMC5298047

[56] Schiller D, Levy I, Niv Y, LeDoux JE, Phelps EA. From fear to safety and back: reversal of fear in the human brain. J Neurosci. 2008;28: 11517–11525. 10.1523/JNEUROSCI.2265-08.2008 18987188 PMC3844784

[57] Koenig S, Uengoer M, Lachnit H. Pupil dilation indicates the coding of past prediction errors: Evidence for attentional learning theory. Psychophysiology. 2017; 10.1111/psyp.13020 29023832

[58] Hygge S, Hugdahl K. Skin conductance recordings and the NaCl concentration of the electrolyte. Psychophysiology. 1985 pp. 365–367. 4011809 10.1111/j.1469-8986.1985.tb01616.x

[59] Bach DR, Daunizeau J, Friston KJ, Dolan RJ. Dynamic causal modelling of anticipatory skin conductance responses. Biol Psychol. Elsevier B.V.; 2010;85: 163–170. 10.1016/j.biopsycho.2010.06.007 20599582 PMC2923733

[60] Burnham KP. Multimodel Inference: Understanding AIC and BIC in Model Selection. Sociol Methods Res. 2004;33: 261–304. 10.1177/0049124104268644

[61] Ohman A, Bjorkstrand PA, Ellstrom PE. Effect of explicit trial-by-trial information about shock probability in long interstimulus interval GSR conditioning. J Exp Psychol. 1973;98: 145–151. 10.1037/h0034313 4704205

[62] Kunimoto M, Kirnö K, Elam M, Karlsson T, Wallin BG. Non-linearity of skin resistance response to intraneural electrical stimulation of sudomotor nerves. Acta Physiol Scand. 1992;146: 385–392. 10.1111/j.1748-1716.1992.tb09433.x 1481693

[63] Courville AC, Daw ND, Touretzky DS. Bayesian theories of conditioning in a changing world. Trends Cogn Sci. 2006;10: 294–300. 10.1016/j.tics.2006.05.004 16793323

[64] O’Reilly JX, Jbabdi S, Behrens TEJ. How can a Bayesian approach inform neuroscience? Eur J Neurosci. 2012;35: 1169–1179. 10.1111/j.1460-9568.2012.08010.x 22487045

[65] Dearden R, Friedman N, Andre D. Model based Bayesian exploration. Proc fifteenth Conf Uncertain Artif Intell. 1999; 150–159. doi: 10.1.1.33.4973

[66] Boll S, Gamer M, Gluth S, Finsterbusch J, Büchel C. Separate amygdala subregions signal surprise and predictiveness during associative fear learning in humans. Eur J Neurosci. 2013;37: 758–767. 10.1111/ejn.12094 23278978

[67] Stephan KE, Penny WD, Daunizeau J, Moran RJ, Friston KJ. Bayesian model selection for group studies. Neuroimage. Elsevier Inc.; 2009;46: 1004–1017. 10.1016/j.neuroimage.2009.03.025 19306932 PMC2703732

[68] Rigoux L, Stephan KE, Friston KJ, Daunizeau J. Bayesian model selection for group studies—Revisited. Neuroimage. Elsevier Inc.; 2014;84: 971–985. 10.1016/j.neuroimage.2013.08.065 24018303

