# Supplementary material for: Human Pavlovian fear conditioning conforms to probabilistic learning
Source: PLoS Comput Biol. 2018 Aug 31;14(8):e1006243. doi: 10.1371/journal.pcbi.1006243 (PMC6118355; doi:10.1371/journal.pcbi.1006243)
Supplement: S1 Fig — (PDF) [file pcbi.1006243.s001.pdf]

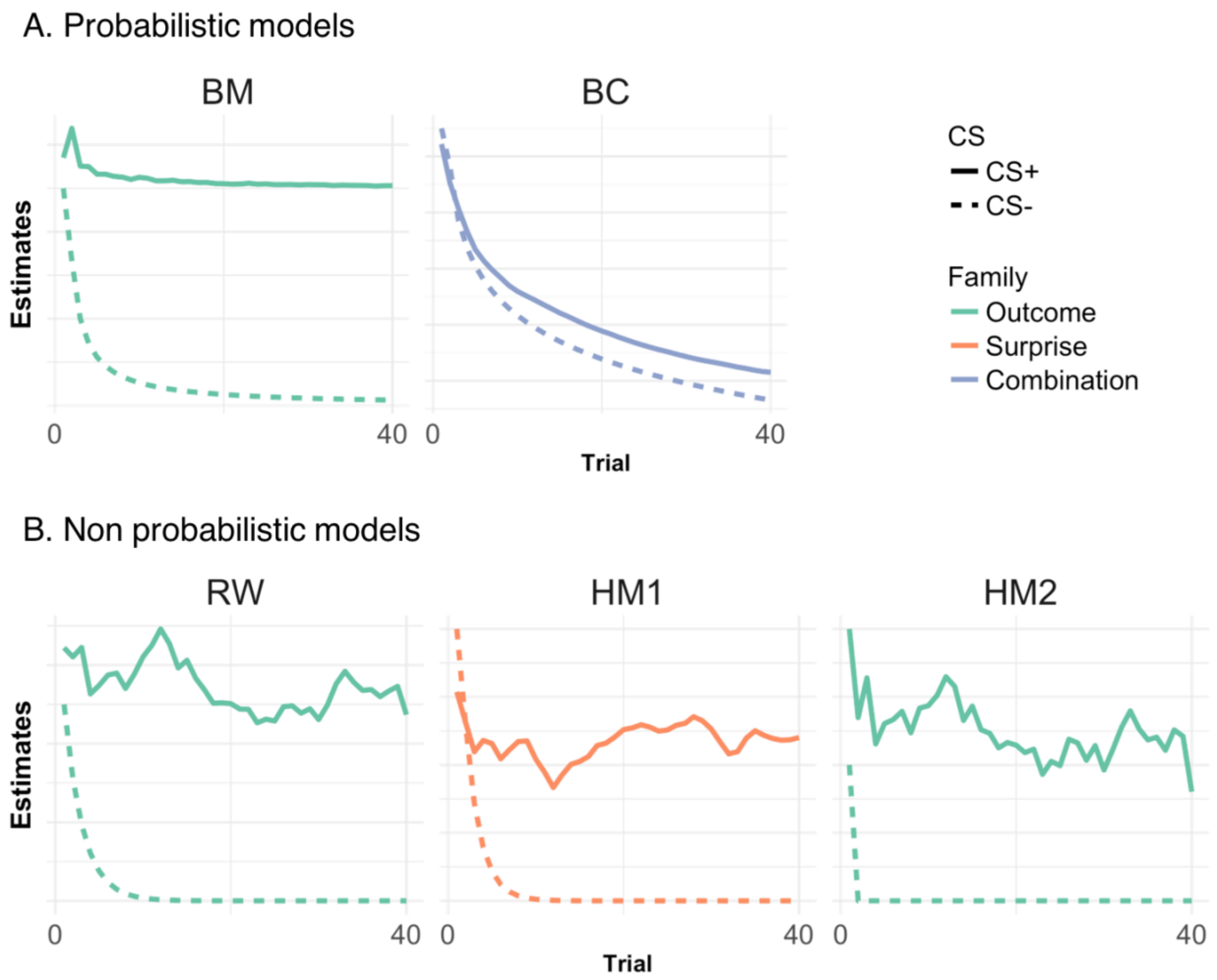

Supplemental Fig 1. Mean model estimates, over 100 simulated CS+/- and reinforcement sequences.
