# Supplementary material for: Human Pavlovian fear conditioning conforms to probabilistic learning
Source: PLoS Comput Biol. 2018 Aug 31;14(8):e1006243. doi: 10.1371/journal.pcbi.1006243 (PMC6118355; doi:10.1371/journal.pcbi.1006243)
Supplement: S2 Fig — (PDF) [file pcbi.1006243.s002.pdf]

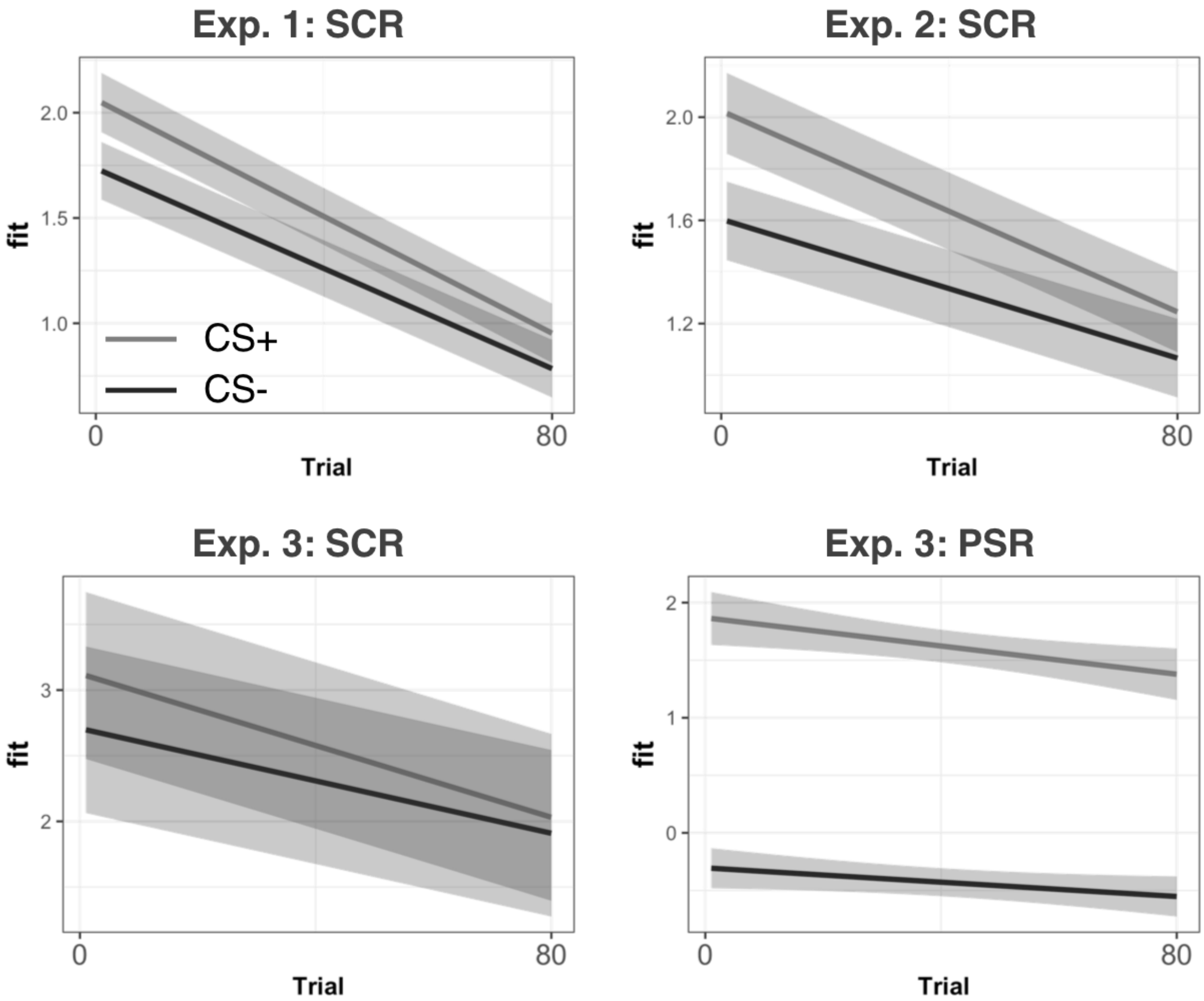

Supplemental Fig 2. Results of LME fitting for SCR and PSR for three experiments, for CS+ / CS- trials. The plotted lines illustrate the fitted slopes across participants.
